# The GSTM1 Null Genotype Increased Risk of Gastric Cancer: A Meta-Analysis Based on 46 Studies

**DOI:** 10.1371/journal.pone.0081403

**Published:** 2013-11-07

**Authors:** Yi Zhao, Xin Deng, Guoqing Song, Shibo Qin, Zhanzhan Liu

**Affiliations:** Department of Pancreato-Breast Surgery, Affiliated Shengjing Hospital of China Medical University, Shenyang, China; Vanderbilt University School of Medicine, United States of America

## Abstract

**Background:**

Glutathione S-transferases M1 (GSTM1) is an important phase II metabolizing enzyme. The null genotype of GSTM1 causes total loss of GSTM1 enzyme activity and numerous studies have investigated the association between GSTM1 null genotype and gastric cancer risk.

**Methods:**

This meta-analysis was designed to investigate the relationship between GSTM1 null genotype and susceptibility to gastric cancer and assess the influence of Helicobacter pylori infection, smoking, Lauren’s classification, and other factors. Odds ratios (ORs) and 95% confidence intervals (CIs) were calculated to estimate the association strength.

**Results:**

A total of 46 eligible studies were indentified and analyzed in this meta-analysis, including 8138 cases of gastric cancer and 13867 controls. Pooled results showed that the GSTM1 null genotype was associated with a significantly increased risk of gastric cancer (OR=1.217, 95% CI: 1.113-1.331, P_heterogeneity_<0.001). Sub-group analysis suggested that the significant association was only observed in Asians (OR=1.273, 95%: 1.137-1.426, P_heterogeneity_ = 0.002), but not in Caucasians. The increased risk was found among H. pylori positive population (OR=1.928, 95% CI: 1.028-3.615, P_heterogeneity_=0.065), while no association was found among H. pylori negative population (OR=0.969, 95% CI: 0.618-1.521, P_heterogeneity_=0.168). For smoking status, the GSTM1 null genotype increased risk of gastric cancer in both ever-smokers and non-smokers. Source of control, sample size, location of tumor and Lauren’s classification did not modify the association.

**Conclusions:**

In this meta-analysis based on 46 epidemiological studies, we show that the GSTM1 null genotype is associated with an increased risk of gastric cancer among Asians but not among Caucasians. H. pylori infection but not smoking status could modify the association.

## Introduction

It has been well demonstrated that gastric cancer is the second leading cause of cancer related death and the fourth most common malignancy, which accounts for 9.7% of total cancer deaths worldwide [1]. As a major public health challenge, it is reported that about one million new cases of gastric cancer were diagnosed in 2008. However, the mechanism of gastric carcinogenesis is still not fully understood. Current evidence suggests that, in combination with environmental factors, low-pentrance susceptibility genes play an important role in the development of cancer [2].

Human glutathione S-transferases (GSTs) are phase II metabolizing enzymes that are critical for protection from cancer by detoxifying numerous potentially cytotoxic or genotoxic compounds [3]. According to their amino acid sequence, immunological cross-reactivity, and substrate specificity, human cytosolic GSTs have been classified into seven families, namely GST alpha, mu, pi, sigma, omega, theta, and zeta [4,5]. Glutathione S-transferase M1 (GSTM1), belonging to the GST mu gene family, is polymorphic and the common deletion polymorphism of GSTM1 has been extensively studied. The homozygous deletion of GSTM1 gene lead to total absence of GSTM1 enzyme activity. It was reported that the deleted GSTM1 genotype was in high percentage of human population, about 40-60% in Europeans [6] and about 50% in Asians [7].

A lot of epidemiological studies have investigated the association of GSTM1 depletion with risk of gastric cancer and several meta-analyses have been performed to clarify this issue [5,8,9]. The most recent meta-analysis was conducted in 2010 [1,6], as well as several other similar meta-analyses and a lot of studies with larger sample size have been published [10-12]. However, limited by number of studies, previous studies failed to assess the influence of some important factors, like Helicobacter pylori infection, the well established risk factor of gastric cancer, smoking status, location of tumor, and sample size [5,8].

Thus, we conducted an updated meta-analysis to comprehensively assess the relationship between GSTM1 deletion polymorphism and risk of gastric cancer and evaluate the influence of confounding factors

## Materials and Methods

### Identification of eligible studies

This study was carried out and reported in agreement with the PRISMA guidelines for systematic reviews and meta-analyses [13] (supplementary information: Checklist S1. PRISMA checklist). Eligible case-control studies were extracted by searching databases and manual search of references of relative articles and reviews. A comprehensive literature search was carried out using electronic databases of PubMed and EMBASE. To avoid selection bias, Chinese databases like China National Knowledge Infrastructure (CNKI) was not searched. The following medical subheadings (MeSH) and key words were utilized during database searching: “glutathione S-transferase M1” or “GSTM1”, “polymorphisms, single nucleotide” or “polymorphism”, and “stomach neoplasms” or “gastric cancer”. Alternative spellings of these key words were also considered. There was no limitation of research and the last research was performed on August 12, 2013. References of related studies and reviews were manually searched for additional studies.

### Inclusion and exclusion criteria

Studies were selected according to the following inclusion criteria: (1) case-control studies; (2) investigating the association between GSTM1 deletion polymorphism and gastric cancer risk; (3) with available genotype distribution data to calculate combined odds ratios (ORs) and 95% confidence intervals (95% CIs). Studies without detail genotype distribution data were excluded. Titles and abstracts of searching records were primarily screened and full text papers were further retrieved to confirm eligibility. Two reviewers (ZY and DX) extracted eligible studies independently according to the inclusion criteria. Disagreement between two reviewers was discussed with another reviewer (SGQ) till consensus was achieved.

### Data extraction

Data of eligible studies was extracted by two reviewers (ZY and DX) independently with a pre-designed data-collection form. The following data was collected: name of first author, year of publication, country where the study was conducted, ethnicity, source of control, number of cases and controls, genotype frequency in cases and controls, H. pylori infection (positive and negative), smoking status (ever-smoker and non-smoker), tumor location (cardia and non-cardia), and Lauren’s classification (diffuse and intestinal). Different ethnicity descents were categorized as Asian, Caucasian, and Latin American. For H. pylori infection and smoking status, we collected data according the original definition of eligible studies and no modification or adjustment was performed. Eligible studies were defined as hospital-based (HB) and population-based (PB) according to the control source. Two reviewers reached consensus on each item.

### Statistical analysis

The association strength between GSTM1 null genotype and gastric cancer risk was measured by OR with 95% CI. The estimated ORs were achieved by pooling genotype distribution data from each eligible study. A 95% CI was used for statistical significance test and a 95% CI without 1 for OR indicating a significant increased or reduced cancer risk. The pooled ORs were calculated for the comparison of null genotype versus present genotype. Subgroup analyses were also conducted to explore the effects of confounding factors: ethnicities, sources of control, sample size, H. pylori infection, smoking status, location, and Lauren’s classification. 

Chi-square based Q test was used to check the statistical heterogeneity between studies, and the heterogeneity was considered significant when p<0.10[14]. Given the significant heterogeneity and to achieve a conservative estimate, random-effects model (based on DerSimonian-Laird method) were used to pool the data from different studies [15]. Meta-regression was performed to detect the source of heterogeneity and a p<0.05 was considered significant[16].

Publication bias was detected with Begg’s funnel plot and the Egger’ linear regression test, and a p < 0.05 was considered significant[17]. To test the influence of publication bias, fail-safe number for p=0.05 (Nfs_0.05_) and p=0.01 (Nfs_0.01_) was also calculated[18].All statistical analyses were calculated with STATA software (version 10.0; StataCorp, College Station, Texas USA). And all P values were two-side.

## Results

**Figure 1:**
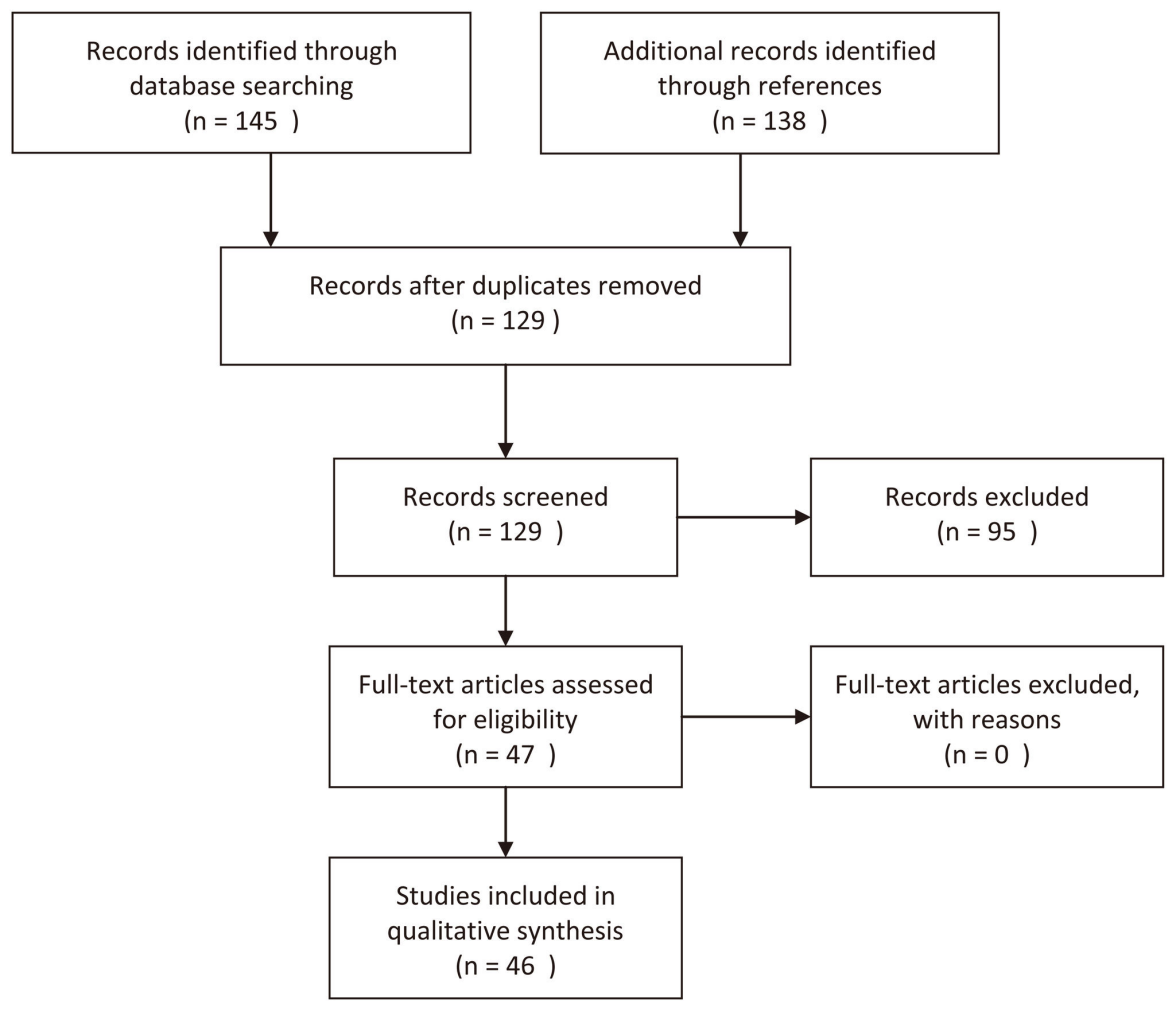
Flow chart of study selection.

The detailed process of study selection was shown in Figure 1. After comprehensive search and rigid selection, 46 eligible studies were identified[10-12,19-61]. The genotype distribution data was available for 8138 patients of gastric cancer and 13867 controls. The baseline characteristics of eligible studies were shown in Table 1. 28 studies were carried out in Asian, 16 studies were in Caucasian, and 2 studies were in Latin American. Notably, 32 of 46 studies were of a small sample size and only 14 studies included more than 500 participants.

**Table 1:** Baseline characteristics of eligible studies.

Author	Year	Country	Ethnicity	Source	Cases	Controls	Case		Control	
							Null	Present	Null	Present
Kato S	1996	Japan	Asian	HB	81	151	30	34	61	59
Katoh T	1996	Japan	Asian	HB	139	126	79	60	55	71
Alves GM	1998	Portugal	Caucasian	HB	148	84	71	77	44	40
Oda Y	1999	Japan	Asian	HB	147	112	91	56	55	57
Cai L	2001	China	Asian	PB	95	94	60	35	43	51
Saadat I	2001	Iran	Asian	PB	42	131	26	16	53	78
Setiawan VW^a^	2001	China	Asian	PB	133	433	40	39	202	205
Shen J	2001	China	Asian	PB	112	675	71	41	361	314
Gao CM	2002	China	Asian	PB	153	223	95	63	133	90
Sgambato A	2002	Italy	Caucasian	HB	8	100	5	3	53	47
Wu MS	2002	China	Asian	HB	356	278	173	183	136	142
Colombo J^a^	2004	Brazil	Latin America	HB	100	150	47	53	62	88
Roth MJ	2004	China	Asian	PB	90	454	24	66	145	309
Suzuki S	2004	Japan	Asian	HB	146	177	87	58	84	93
Torres MM	2004	Colombia	Latin America	HB	46	96	30	16	36	60
Lai KC	2005	China	Asian	HB	123	121	73	50	55	66
Li H	2005	Chian	Asian	HB	102	62	67	33	26	36
Nan HM	2005	Korea	Asian	HB	110	220	73	34	130	90
Palli D	2005	Italy	Caucasian	PB	175	546	90	85	275	271
Shen J	2005	China	Asian	PB	114	693	71	41	361	314
Tamer L	2005	Turkey	Caucasian	HB	70	204	40	30	88	116
Agudo A	2006	UK	Caucasian	PB	243	946	122	120	498	434
Hong SH	2006	Korea	Asian	HB	108	238	60	48	134	104
Lee K	2006	Chile	Caucasian	HB	73	263	13	60	56	207
Martínez C	2006	Spain	Caucasian	PB	98	329	33	54	149	180
Boccia S	2007	Italy	Caucasian	HB	102	254	59	43	135	119
Ruzzo A	2007	Italy	Caucasian	HB	126	144	35	44	61	51
Wideroff L	2007	USA	Caucasian	PB	116	209	61	55	121	87
Tripathi S^a^	2008	India	Asian	HB	76	100	31	45	39	61
Al-Moundhri MS	2009	Oman	Caucasian	HB	107	107	42	65	32	75
Malik MA	2009	India	Asian	HB	108	195	64	44	79	116
Masoudi M	2009	Iran	Caucasian	PB	67	134	37	30	60	74
Moy KA	2009	China	Asian	PB	307	911	98	72	415	320
Piao JM	2009	Korea	Asian	PB	2213	1699	1225	988	923	776
Zendehdel K	2009	Sweden	Caucasian	PB	126	471	70	54	239	230
Nguyen TV	2010	Vietnam	Asian	HB	59	109	43	16	75	34
Palli D	2010	Italy	Caucasian	PB	314	548	166	130	275	271
Yadav DS	2010	India	Asian	HB	133	270	49	84	120	150
Darazy M	2011	Lebanese	Caucasian	PB	13	70	6	7	12	58
Luo YP	2011	China	Asian	PB	123	129	93	50	71	58
Yadav D	2011	India	Asian	PB^b^	41	130	11	30	38	92
Zhang AP	2011	China	Asian	PB	194	412	105	89	194	218
García-González MA	2012	Spain	Caucasian	PB	557	557	284	273	267	290
Jing C	2012	China	Asian	HB	410	410	240	170	207	203
Kim HJ	2012	Korea	Asian	HB	102	200	61	41	124	76
Malakar M	2012	India	Asian	PB	102	204	57	45	97	107

PB: population-based; HB: hospital-based; a: only genotype data of healthy controls were extracted; b: source of controls were not described and the study was assumed as PB

### Overall analysis

**Figure 2:**
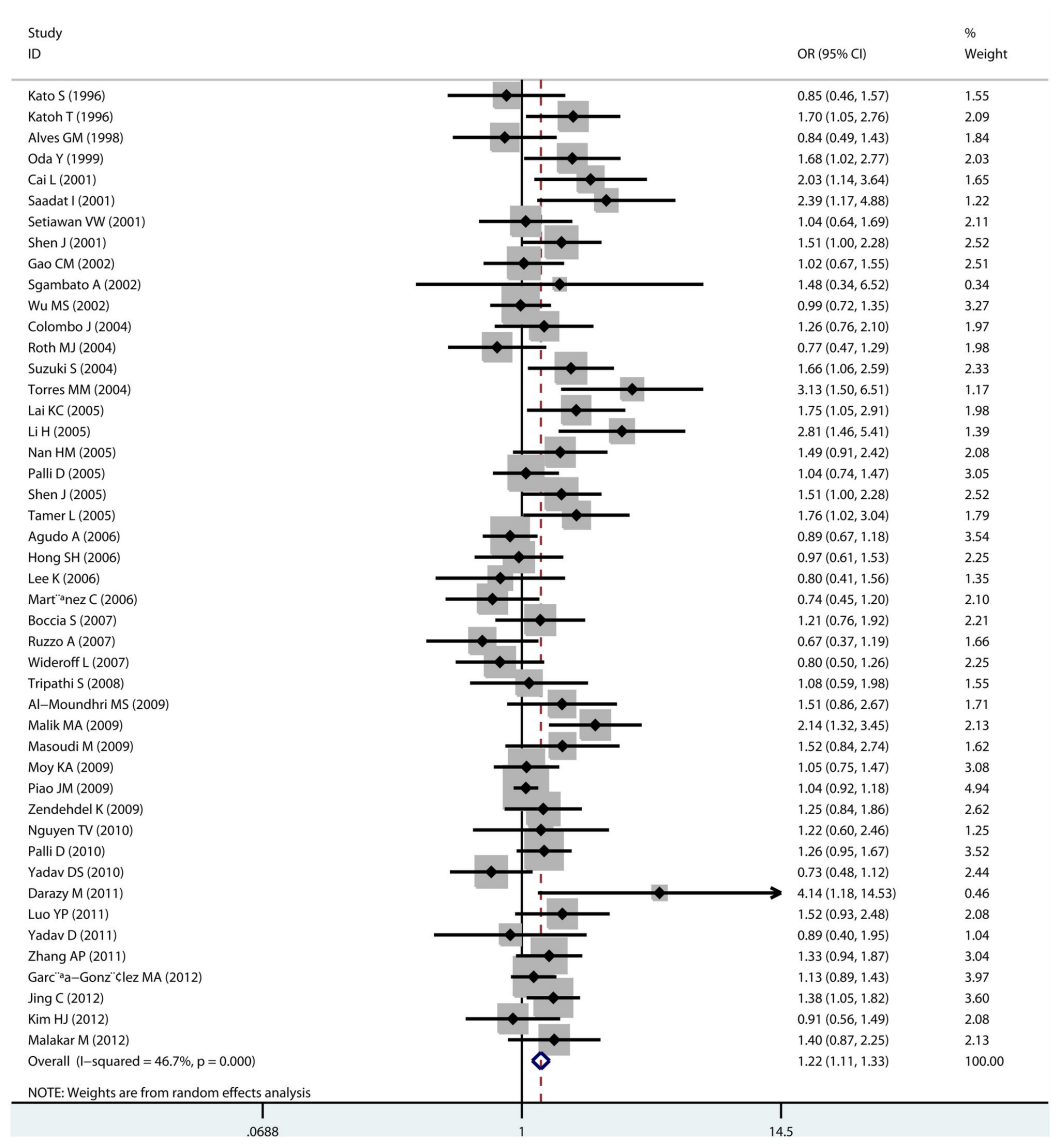
Overall analysis of GSTM1 null genotype and gastric cancer risk. A number of 46 studies were included.

**Figure 3:**
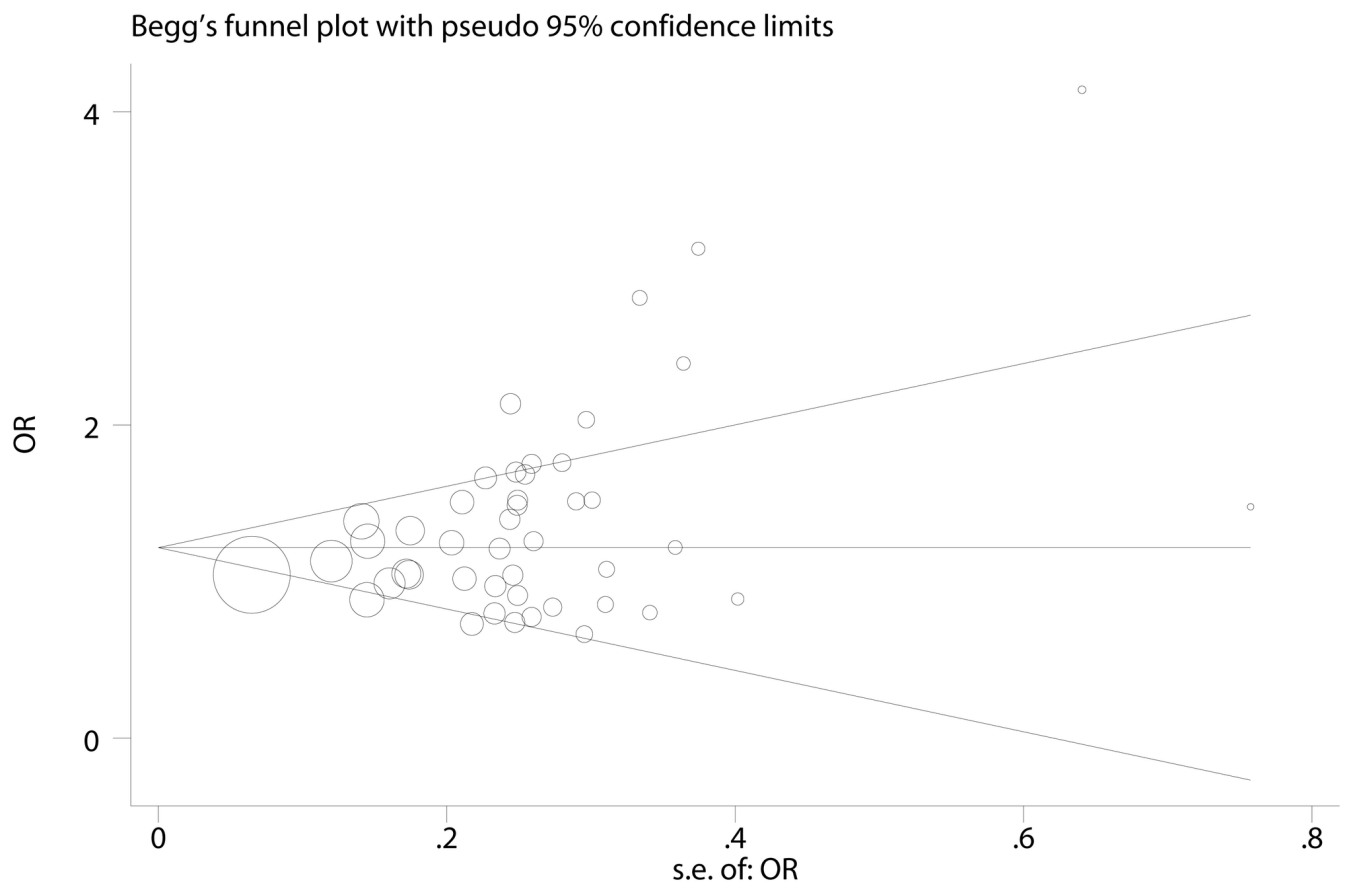
Funnel plot for the overall analysis of GSTM1 null genotype and gastric cancer risk. Circles represent the weight of each study.

All meta-analysis results were shown in Table 2. By pooling all 46 eligible studies, we found the GSTM1 null genotype was associated with a significantly increased risk of gastric cancer (OR=1.217, 95% CI: 1.113-1.331, P_heterogeneity_<0.001; Figure 2). Since significant heterogeneity existed, meta-regression was performed to detect the source of heterogeneity and the results suggested that ethnicities (p<0.001), source of control(p<0.001), and sample size (p<0.001) contributed to the heterogeneity. Egger’s test (p=0.02) and Begg’s test (p=0.003) found the evidence of publication bias (Figure 3). However, the fail-safe-number was large (Nfs_0.05_=1299.9, Nfs_0.01_=602.8), which suggested that the publication bias was feeble and our result is solid.

**Table 2:** Meta-analysis results of GSTM1 polymorphism and gastric cancer risk.

comparison	No. of Studies	OR (95% CI)	Heterogeneity
Overall	46	1.217 (1.113-1.331)*	p<0.001
Source of Control			
Hospital-Based	24	1.283 (1.104-1.490) *	0.002
Population-Based	22	1.156 (1.041-1.284) *	0.041
Enthnicity			
Asian	28	1.273 (1.137-1.426) *	0.002
Caucasian	16	1.081 (0.941-1.243)	0.094
Latin American	2	1.906 (0.784-4.630)	0.046
Sample Size			
Small	32	1.296 (1.125-1.494) *	p<0.001
Large	14	1.120 (1.029-1.220) *	0.311
Smoking Status			
Non-smoker	12	1.777 (1.301-2.426) *	p<0.001
Ever-smoker	11	1.459 (1.024-2.077) *	0.014
H. pylori Infection			
Positive	3	1.928 (1.028-3.615) *	0.065
Negative	4	0.969 (0.618-1.521)	0.168
Location of Tumor			
Cardia	3	0.904 (0.648-1.261)	0.338
Non-Cardia	2	1.051 (0.831-1.331)	0.394
Lauren’s Classification			
Diffuse Type	5	1.162 (0.776-1.741)	0.066
Intestinal Type	5	1.524 (0.998-2.327)	0.017

OR: odds ratio, 95% CI: 95% confidence intervals; p<0.1 indicates significant heterogeneity; * significant association

### Sub-group analysis

#### Ethnicities

Sub-group analysis for ethnicities was performed and the increased risk of gastric cancer was only observed in Asians (OR=1.273, 95%: 1.137-1.426, P_heterogeneity_=0.002), while no significant association was found in Caucasians or Latin Americans (Figure S1). 

#### Source of control

The results showed that source of controls did not affect the pooled results and we observed a significantly increased risk of gastric cancer both in PB and HB studies.

#### Sample size

As shown in Table 1, most eligible studies about GSTM1 polymorphism and gastric cancer were small-sized (less than 500 participants). The mean number of participants for “small studies” was 252 and the mean for “large studies” was 964. Sub-group analysis revealed that the pooled results did not differ between large studies and small studies, since increased susceptibility was observed in both sub-groups (Table 2).

#### Helicobacter pylori infection

HP infection is a well known risk factor of gastric cancer and 4 studies provided data about HP infection status and GSTM1 genotype distribution. As shown in Table 2, the null genotype of GSTM1 was associated with an elevated risk of gastric cancer in the HP positive sub-group (OR=1.928, 95% CI: 1.028-3.615, P_heterogeneity_=0.065), while no significant association was found in the HP negative sub-group (OR=0.969, 95% CI: 0.618-1.521, P_heterogeneity_=0.168).

#### Smoking status

**Figure 4:**
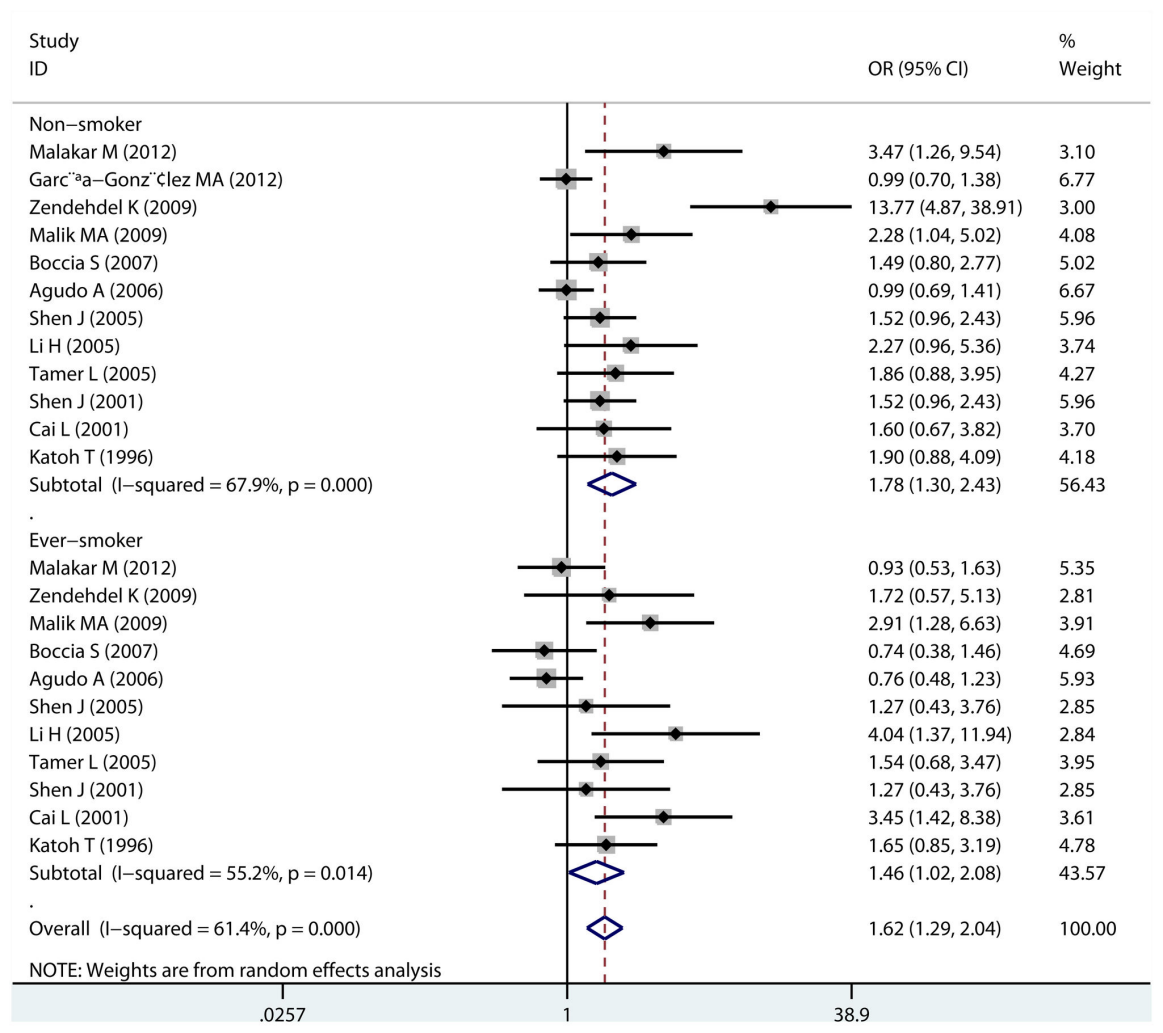
Sub-group analysis of smoking status (ever-smoker and non-smoker).

Smoking is a risk factor of various kinds of cancer, including gastric cancer, and GST family is also involved in the metabolism of various carcinogens in cigarette smoke. As shown in Table 2, data of smoking status and GSTM1 genotype distribution were available in 12 studies. Sub-group analysis results suggested that there was no difference of gastric cancer risk between ever-smokers and non-smokers, because significantly increased risk was found in both sub-groups (Figure 4).

#### Location and Lauren’s classification

We also performed stratified analyses according to location of tumor (cardia and non-cardia) and Lauren’s classification (diffuse and intestinal). The number of studies available for the sub-group analysis of location was quite small (3 studies), and no significant association of GSTM1 null genotype with gastric cancer risk was observed in neither sub-group (Table 2). As for sub-groups of Lauren’s classification, we did not found any significant association for diffuse type cancer or intestinal type cancer.

## Discussion

Genetic polymorphisms are natural DNA sequence variations and the expected frequency is about 1% among healthy population [62]. Functional genetic polymorphism in the gene regulation region or coding sequences could change gene expression or function. Additionally, genetic polymorphism may, to some degree, explain the inter-individual variation and diversity, and has been recently considered as principal genetic elements involved in the development of cancer [63]. The GST gene family encoding phase II detoxification enzymes is critical for the protection against various chemical carcinogenesis [3]. The GSTM1 enzyme is responsible for the metabolism of reactive electrophilic intermediates, including environmental pollutants and other polycyclic aromatic hydrocarbons, which are potent carcinogenic agents. Thus, impaired GSTM1 function may lead to serious DNA damage and carcinogenesis. Considering that the GSTM1 null genotype caused a complete loss of GSTM1 enzyme activity, it is biologically plausible that the GSTM1 null genotype may increase risk of gastric cancer.

Since the first study in 1991 by Strange and colleagues [64] which reported the association between the GSTM1 null genotype and increased risk of gastric cancer, a lot of epidemiological studies about the relationship between GSTM1 and gastric cancer have been conducted[23,48,55]. Limited by number of studies, the conclusion about GSTM1 null genotype and gastric cancer was still unclear, as well as influence of some important factors like H. pylori infection and smoking status. Since a large number of studies have been published [10-12], it is necessary to perform an update meta-analysis to assess the association between GSTM1 and gastric cancer and explore the effect of H. pylori infection, smoking, location and Lauren’s classification.

In this study, we identified 46 eligible studies, including 8138 gastric cancer cases and 13867 controls, which could provide sufficient statistic power. By pooling all available data, we found the null genotype was associated with a statistically elevated risk of gastric cancer (OR=1.217, 95% CI: 1.113-1.331), which was consistent with previous meta-analyses [8,65]. By stratifying studies according to ethnicities, increased risk of gastric cancer was only observed in Asians and no significant association was found in Caucasians or Latin Americans, which was also in agreement with previous studies. The ethnic difference was common for genetic association studies, which may be due to different genetic background and environmental differences. Additionally, the incidence of gastric cancer is quite heterogeneous in Asia population, and the findings should be explained with caution when applied to a specific area. Notably, heterogeneity was significant in this meta-analysis (Table 2). Meta-regression analysis indicated that ethnicities (p<0.001), source of control (p<0.001), and sample size (p<0.001) were the source of heterogeneity. For source of control, participants from hospital may have different genetic background compared with those from general population. To achieve an acute estimation of the relationship between GSTM1 null genotype and gastric cancer risk, future studies should take these factors into consideration. 

Helicobacter pylori, the group I carcinogen classified by World Health Organization, is one of the most important risk factors for gastric cancer[66]. By performing sub-group analysis, we only found the increased risk of gastric cancer in the H. pylori positive group (OR=1.928, 95% CI: 1.028-3.615), while there was no significant association in the H. pylori negative group (OR=0.969, 95% CI: 0.618-1.521). This finding suggested that H. pylori infection could modify the association between GSTM1 polymorphism and susceptibility to gastric cancer [67-69]. Tobacco smoke contains various carcinogens like N-nitrosamines, polycylic aromatic hydrocarbons, and heterocyclic amines, which require detoxification by different pathways, including GSTs. To assess the influence of smoking, we extracted data from 12 eligible studies and found that smoking status did not alter the relationship between GSTM1 null genotype and risk of gastric cancer. This may be explained by that GSTM1 is just a member of GST family and the null genotype will not significantly impair the overall GST enzyme activity. For location of tumor and Lauren’s classification, we did not found any significant association. Given that studies included in these sub-groups were few (Table 2), further studies are warranted.

Compared with previous meta-analysis, we included more studies and performed sub-group analyses to assess the influence of ethnicities, source of controls, sample size, H. pylori infection, smoking, tumor location, and Lauren’s classification. Notably, we searched databases of PubMed and EMBASE but not China National Knowledge Infrastructure (CNKI), because CNKI Chinese-language database, which is usually not accessible for non-Chinese researchers. However, limitations of this meta-analysis should be highlighted. First, Egger’s test and Begg’s test suggested the evidence of publication bias. We calculated the fail-safe number and the number was large enough (Nfs_0.05_=1299.9, Nfs_0.01_=602.8) to provide credence to our results. Secondly, heterogeneity was significant in this study. To achieve a precise and conservative estimation, we used random-effects model to pool eligible studies and meta-regression found that ethnicities, source of control, and sample size were the source of heterogeneity. Thirdly, in the sub-group analysis of location, number of studies was relatively small and the results should be interpreted with caution.

To summary, in this meta-analysis based on 46 epidemiological studies, we show that the GSTM1 null genotype is associated with increased risk of gastric cancer among Asians but not among Caucasians. The null genotype increased susceptibility to gastric cancer both in ever-smokers and non-smokers, while the significant association was only observed in H. pylori positive population.

## Supporting Information

Checklist S1 **PRISMA checklist.**
(DOC)

Figure S1 **Sub-group analysis of ethnicities.**
(TIF)
