# Supplementary figures and images for: The GSTM1 Null Genotype Increased Risk of Gastric Cancer: A Meta-Analysis Based on 46 Studies
Source: PLoS One. 2013 Nov 7;8(11):e81403. doi: 10.1371/journal.pone.0081403 (PMC3820558; doi:10.1371/journal.pone.0081403)

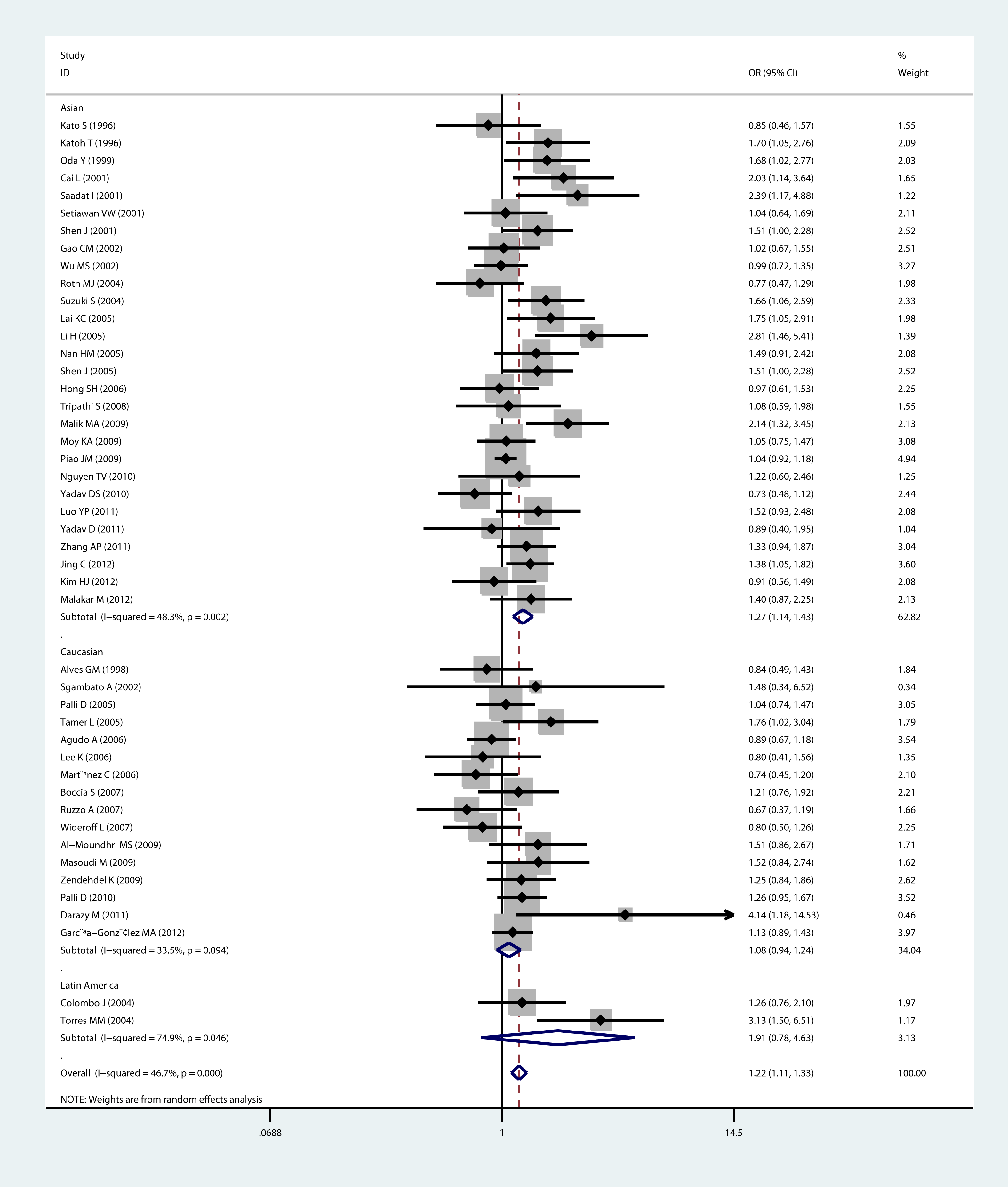

Supplement: Figure S1 — Sub-group analysis of ethnicities. (TIF) [file pone.0081403.s002.tif]
